# A recently introduced *Dichelobacter nodosus* strain caused an outbreak of footrot in Norway

**DOI:** 10.1186/1751-0147-56-29

**Published:** 2014-05-13

**Authors:** Marianne Gilhuus, Bjørg Kvitle, Trine M L’Abée-Lund, Synnøve Vatn, Hannah J Jørgensen

**Affiliations:** 1 Norwegian Veterinary Institute, P.O. Box 750, Sentrum N-0106, Oslo, Norway; 2 Department of Food Safety and Infection Biology, Norwegian University of Life Sciences (NMBU), Campus Adamstuen, P.O. Box 8146 Dep, N-0033 Oslo, Norway; 3 Animalia – Norwegian Meat and Poultry Research Center, P.O. Box 396, Økern N-0513, Oslo, Norway

**Keywords:** Cow, Genotyping, Goat, Pulsed-field gel electrophoresis, PFGE, Serogroup converison, Sheep

## Abstract

**Background:**

In 2008, an outbreak of ovine footrot occurred in Norway. *Dichelobacter nodosus* isolates collected between 2008 and 2011 have been characterised. Isolates defined as virulent by the gelatin gel test (GG-test) were only found in sheep in Rogaland County, where the severe cases of footrot were registered. The majority (96%) of the virulent isolates belonged to serogroup A. It is suspected that they represent a newly introduced strain, and the aim of the present study was to investigate whether they are genetically similar. Sixty-one virulent isolates from sheep and 116 benign isolates from sheep, cattle and goats were included. Four GG-test virulent isolates from Danish sheep were also included. All isolates were genotyped by pulsed-field gel electrophoresis (PFGE) and by PCR for *pgr* variant determination.

**Results:**

The Norwegian virulent isolates were assigned to 8 pulsotypes (PTs), while the benign isolates were assigned to 66 PTs. Thirty-seven (68.5%) of the 54, virulent, serogroup A isolates belonged to the same PT, and included isolates from 2008 through 2011. Isolates belonging to this PT were defined as the outbreak strain. The remaining virulent serogroup A isolates belonged to 4 PTs differing by ≤3 bands from the outbreak strain. Two virulent, Danish, serogroup A isolates differed by 2 bands from the Norwegian outbreak strain. All but 3 (95%) of the virulent isolates had the *pgrA* variant while 85% of the benign isolates had the *pgrB* variant.

**Conclusion:**

This study provides evidence that the footrot outbreak in Norway in 2008 most likely was caused by new introduction and local spread of one virulent *D. nodosus* strain.

## Background

In 2008, footrot was diagnosed in Norwegian sheep for the first time in 60 years [1]. The infection is caused by *Dichelobacter nodosus*[2], and clinical signs range from interdigital dermatitis to complete loosening of the hoof horn. The severity of lesions depends on the virulence of the infecting bacterial strain, climatic conditions and the breed of sheep [3].

Following the outbreak, *D. nodosus* isolates from sheep, cattle and goats collected between 2008 and 2011 from different geographic locations in Norway were characterised with respect to virulence and serogroup [4]. Isolates that produced heat stable proteases, as shown by the gelatin gel test (GG-test) [5], were defined as virulent and were only found in sheep in the county of Rogaland where the most severe cases of footrot were registered. Interestingly, 96% of these isolates belonged to serogroup A [4]. Isolates defined as benign showed greater serogroup diversity and were more spread geographically [4]. There was also an association between laboratory-defined virulence and clinical manifestations in sheep [6,7]. An ongoing programme to control and eradicate ovine footrot, caused by virulent *D. nodosus*, was initiated in Norway in 2009 [8].

It is believed that the Norwegian footrot outbreak was caused by introduction and local spread of a virulent *D. nodosus* strain belonging to serogroup A. To confirm this it was necessary to genotype the isolates. It is possible that the infection was introduced through import of sheep. Import of live animals to Norway is restricted, but a few sheep imports were registered in the years prior to the outbreak in 2008, mainly from Denmark [9].

A few studies have used genotyping for molecular epidemiological investigations of *D. nodosus*[10-13]. Pulsed field gel electrophoresis (PFGE) is discriminatory and reproducible, and has been used for typing *D. nodosus*[10,13]. Typing based on the proline-glycine repeat (*pgr*) gene in *D. nodosus* was described by Calvo-Bado *et al*. [14], who developed a PCR assay to discriminate between the two *pgr* variants *pgrA* and *pgrB*.

In this study, a collection of *D. nodosus* isolates were genotyped by PFGE and *pgr* typing to find out whether the GG-test virulent, serogroup A isolates are genetically similar. The aim was to confirm whether the footrot outbreak in Norway was caused by local spread of one, single virulent *D. nodosus* strain.

## Methods

### Bacterial isolates

A total of 177 *D. nodosus* isolates from Norwegian sheep, cattle and goats were investigated (Table 1). The isolates were collected between 2008 and 2011, from 12 of the 19 Norwegian counties and 131 different farms. One hundred and thirty-one of the isolates were from Rogaland County, where the most severe cases of footrot had occurred.

**Table 1:** ***Dichelobacter nodosus *** **isolates from Norway included in the study, with respect to host species, virulence, number of farms and *fim*** **A variant (serogroup A-M)**

	**No. of isolates**	**No. of farms**	**A**	**B**	**C**	**D**	**E**	**F**	**G**	**H**	**I**	**M**
Virulent isolates sheep	61	52^a^	54	1	1	0	2	0	0	2	1	0
Benign isolates sheep	79	68^a^	26	13	21	0	2	0	5	8	4	0
Benign isolates cattle	34	16	11	8	3	1	4	0	2	2	1	2
Benign isolates goats	3	2	1	2	0	0	0	0	0	0	0	0
Total	177	131^a,b^	92	24	25	1	8	0	7	12	6	2

^a^From 4 sheep flocks both virulent and benign isolates were included.

^b^From 3 farms isolates from both sheep and cattle were included.

One hundred and fifty-five of the isolates have been described previously [4] and were selected to represent all Norwegian farms from which *D. nodosus* had been cultured before the end of 2011. Twenty-eight of them were from 10 farms that had been sampled 2–3 times with 3–15 month intervals and where isolates from all sampling occasions were included.

Twenty-two of the isolates were collected in a recent study of infectious foot diseases in Norwegian cattle [15,16].

All the isolates had been confirmed as *D. nodosus* and characterised with respect to virulence and serogroup as described previously [4] (Table 1). Isolates shown to produce heat stable or heat labile proteases by the GG-test were defined as virulent and benign, respectively, regardless of the clinical manifestations in the animals.

The 61 virulent Norwegian isolates included in the study were all from sheep in Rogaland County. Fifty-four of them belonged to serogroup A. The 116 benign isolates were from sheep, cattle and goats and 12 different counties.

Four isolates from 3 Danish sheep flocks were also included. These isolates were collected in 2009 [17] and had also been characterised as described previously [4]. All 4 isolates were virulent and belonged to serogroup A (n = 2) or E (n = 2).

### Pulsed-field gel electrophoresis

All isolates were analysed by PFGE essentially as described by Buller *et al*. [10], but with the following modifications: Isolates were cultured on 2% TAS (trypticase-arginine-serine) agar for 2–3 days, and suspended in 300 μl wash buffer [10]. The agarose plugs were washed 5 times in Tris-EDTA (TE) buffer. Prior to restriction, plugs were washed twice with 0.6 ml TE at 25°C for 20 min. DNA was restricted with 50 U of *Apa*I (Sigma-Aldrich, St Louis, MO) in a 100-μl volume containing restriction buffer and 0.1 mg/ml Bovine serum albumin (BSA) (Sigma-Aldrich) for 3 h at 30°C. An additional 10 U *Apa*I was added halfway through the incubation period. The reference strain ATCC® 27521 was prepared as described above and included on each gel. Lambda ladder (New England Biolabs, Ipswich, MA) was used as size standard. Restriction fragments were separated in a CHEF-DR® III electrophoresis system (Bio-Rad, Hercules, CA) at 6 V/cm, with a pulse time of 0.2 to 25 s for 24 h.

Differentiation of banding patterns was performed using BioNumerics (version 6.1; Applied Maths, Kortrijk, Belgium) and visual inspection. PFGE bands larger than the smallest ladder fragment (48.5 kb) were included in the analysis. A unique banding pattern was defined as a pulsotype (PT). Pairwise similarity coefficients were calculated using the Dice formula, and the dendrogram was created using the unweighted pair group method with arithmetic averages. Optimisation and position tolerance settings were set at 1.5.

### PCR for *pgr*

All isolates were tested for the presence of *pgrA* and *pgrB* by PCR as described by Calvo-Bado *et al*. [14]. DNA was extracted from 48 h HEPES-TAS broth cultures using a nucliSENS® easyMAG™ extractor (bioMèrieux, Boxtel, The Netherlands) following the manufacturers’ instructions. Extracted DNA was stored at −20°C.

For the PCR, HotStarTaq Master Mix (QIAGEN GmbH, Hilden, Germany) was used with 5 μM of each primer. Amplification was performed on an MJ Research DNA Engine Dyad® (Bio-Rad) with an initial denaturation step of 95°C for 2 min followed by 31 cycles of 95°C for 1 min, 60°C (*pgrA*) or 55°C (*pgrB*) for 1 min, 72°C for 2 min, and a final extension time of 72°C for 7 min. PCR products were analysed by electrophoresis in a 1% agarose gel stained with GelRed™ (Biotium, Hayward, CA).

DNA from control strains of *D. nodosus* were kindly provided by Dr. L.A. Calvo-Bado, Department of Biological Sciences, University of Warwick, Coventry, UK: VCS1703A as positive control for *pgrA*, and C305 as positive control for *pgrB*.

### Statistical analyses

Descriptive statistics were performed using Microsoft Excel 2010 (Microsoft Corporation, Redmond, WA). Further statistical analyses were performed using GraphPad Prism version 6.00 for Windows (GraphPad Software, La Jolla, CA).

Fisher´s exact test [18] was used to investigate whether there was a difference between isolates defined as benign *versus* isolates defined as virulent with respect to the proportions of isolates having the *pgrA* or *pgrB* gene. Results were considered significant when p < 0.05.

## Results

### PFGE

**Figure 1:**
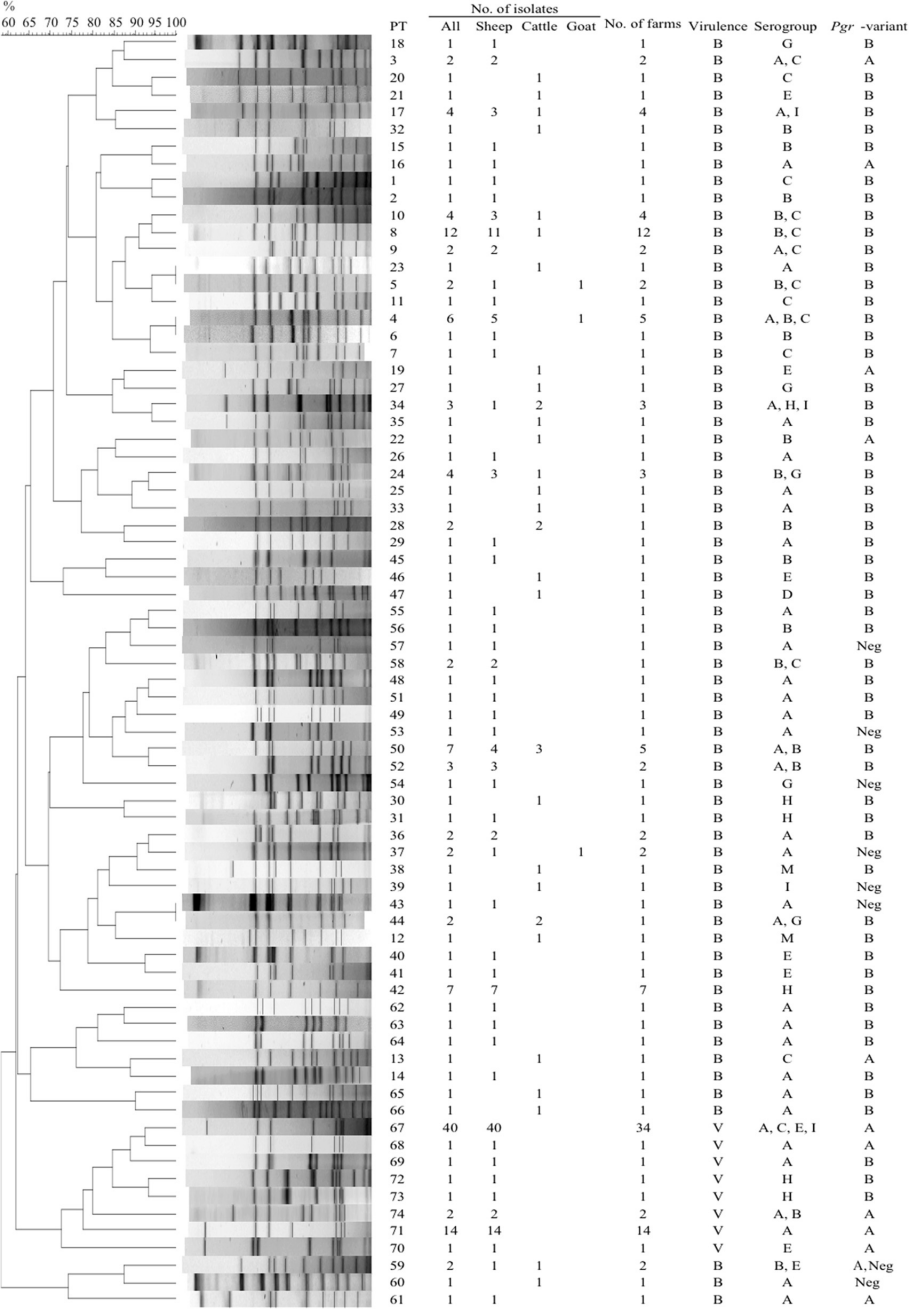
**Dendrogram of *Dichelobacter nodosus *isolates from Norway analysed by Pulsed-field gel electrophoresis (PFGE), including one isolate from each pulsotype (PT).** The assigned PT number and the total number of isolates assigned to each PT are indicated to the right, and for each PT the number of isolates from each species, number of farms from which they were collected, and the virulence, serogroup and proline-glycine repeat (*pgr*) variant of the isolates are listed.

The 177 Norwegian *D. nodosus* isolates were divided into 74 different pulsotypes (PTs), consisting of 5–11 bands. Between 1 and 40 isolates were assigned to each PT. The benign isolates were assigned to 66 PTs that clustered with >58% similarity and virulent isolates were assigned to 8 PTs that clustered with >72% similarity (Figure 1). Virulent and benign isolates belonged to different PTs.

The 54 virulent, serogroup A isolates were divided into 5 PTs (Table 2), and the majority (n = 37) belonged to PT 67. This PT included isolates collected from 2008 through to 2011. The other virulent, serogroup A isolates belonged to 4 different PTs that differed by ≤3 bands from PT 67 (Table 2). Three of the 7 virulent, non-serogroup A isolates also belonged to PT 67, while the remaining 4 isolates belonged to 4 PTs that differed from PT 67 by 2–4 bands.

**Table 2:** **The five pulsotypes (PT) to which the Norwegian, virulent, serogroup A *Dichelobacter nodosus *** **isolates belonged with respect to the band differences from PT 67, and the distribution of isolates with respect to the year of collection**

**PT**	**Band diff. from PT67**	**No. of isolates**				
		**Total (%)**	**2008**	**2009**	**2010**	**2011**
67	-	37 (68.5)	1	12	16	8
68	1	1 (1.9)	0	0	1	0
69	2	1 (1.9)	0	0	1	0
71	2	14 (25.9)	0	0	10	4
74	3	1 (1.9)	0	0	1	0

Twenty-one PTs included more than one isolate (Figure 1). Thirteen of these were only found in one county. Sixteen PTs included isolates belonging to different serogroups (Figure 1). Isolates from sheep (n = 140), cattle (n = 34) and goats (n = 3) were divided in to 52, 29 and 3 PTs, respectively. Twenty-six (18.6%) isolates from sheep and 10 (29.4%) isolates from cattle belonged to 7 PTs common to both species (Figure 1). The PTs of the 3 isolates from goats were all common to PTs from sheep.

**Figure 2:**
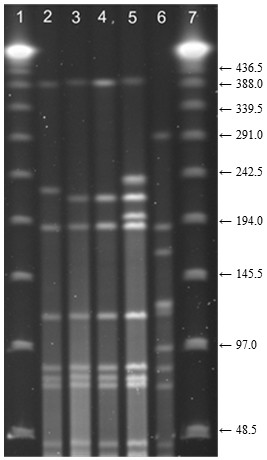
**Pulsed-field gel electrophoresis (PFGE) of *Apa*I restricted, virulent *Dichelobacter nodosus *isolates belonging to serogroup A from Norway and Denmark.** Lanes: 1 and 7, lambda ladder molecular size marker; 6, *D. nodosus* ATCC® 27521 reference strain; 2 and 5, isolates from Denmark; 3–4, isolates from Norway. Numbers to the right indicate molecular size (kilobases).

The 4 Danish isolates were assigned to different PTs. The 2 serogroup A isolates belonged to PTs that differed from PT 67 by 2 bands (Figure 2). The 2 serogroup E isolates belonged to PTs that differed from PT 67 by 4 bands.

From 6 of the 10 farms from where isolates had been collected on 2–3 occasions, isolates from all samplings belonged to the same PT.

**Figure 3:**
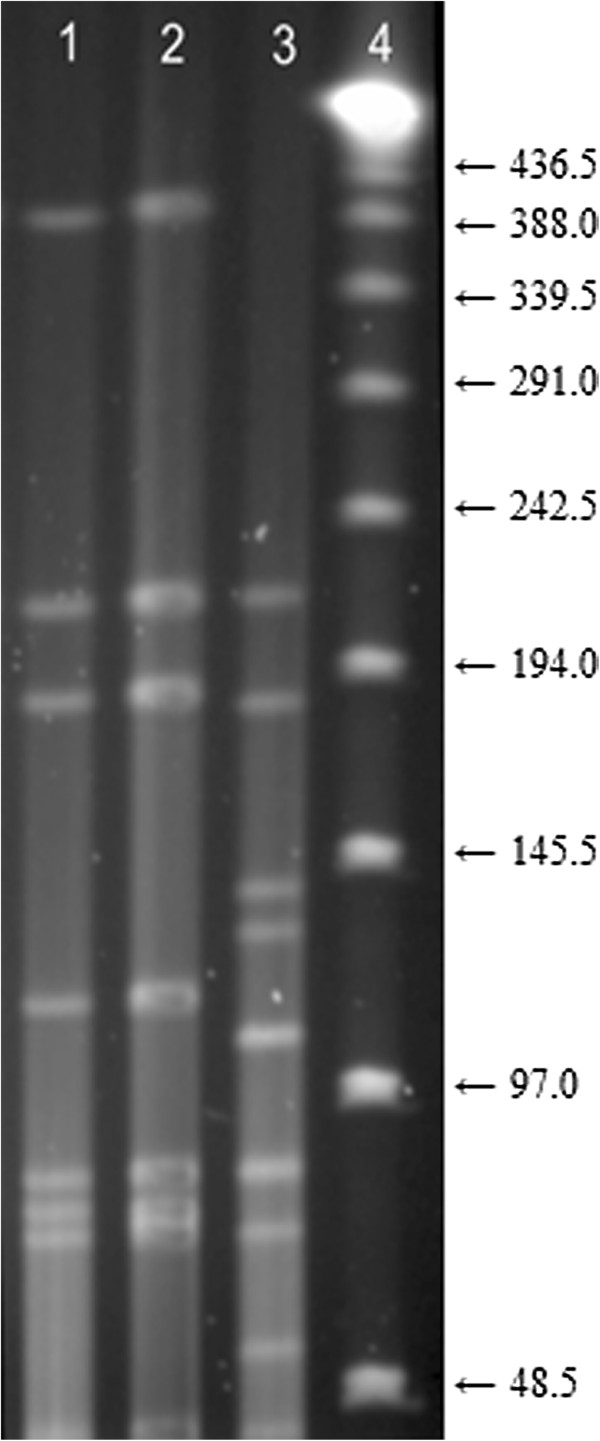
**Pulsed-field gel electrophoresis (PFGE) of three *Apa*I restricted *Dichelobacter nodosus *isolates from one farm indicating a possible case of serogroup conversion.** Lanes: 4, lambda ladder molecular size marker; 1, virulent, serogroup A isolate; 2, virulent, serogroup C isolates; 3, benign, serogroup C isolate. Numbers to the right indicate molecular size (kilobases).

The 3 virulent, non-serogroup A isolates assigned to PT 67 were from 3 farms where a virulent, serogroup A isolate, belonging to PT 67 had also been cultured. It is possible that this represent cases of serogroup conversion. From one of the farms, a virulent, serogroup A isolate, a virulent, serogroup C isolate and a benign, serogroup C isolate were available for investigation. The 2 virulent isolates were identical (PT 67) while the benign isolate differed (Figure 3). No benign isolates were available from the 2 other farms.

### PCR for *pgr*

Results from the *pgr* PCR are presented in Table 3. The *pgrA* variant was detected in 53 of the 54 virulent, serogroup A isolates and in 5 of the 7 virulent, non-serogroup A isolates. Five per cent of the isolates were negative for both *pgrA* and *pgrB*, with no other common features than being benign. The association between virulence, as defined by the GG-test, and *pgr* variant was statistically significant.

**Table 3:** **Distribution of *pgr *** **variant among virulent and benign isolates of *Dichelobacter nodosus *** **from Norway**

	***pgrA *** **(%)**	***pgrB *** **(%)**	***pgrA*/*pgrB negative *** **(%)**
Virulent isolates	58 (95.1)	3 (4.9)	0 (−)
Benign isolates	8 (6.9)	99 (85.3)	9 (7.8)
Total	66 (37.3)	102 (57.6)	9 (5.1)

With only one exception, isolates within a PT had the same *pgr* variant (Figure 1). Three virulent isolates were *pgrB*-positive. They belonged to serogroup A (n = 1) or H (n = 2) and differed in pulsotype from PT 67 by 2 and 4 bands, respectively.

In the farm with a possible case of serogroup conversion, the 2 virulent isolates belonging to serogroup A and C both had the *pgrA* variant, while the benign serogroup C isolate had the *pgrB* variant.

The 4 virulent Danish isolates were all *pgrA*-positive.

## Discussion

This study shows that the *D. nodosus* population in Norway, as a whole, is genetically diverse, but that isolates defined as virulent by the GG-test, belonging to serogroup A are genetically similar. It provides evidence that the outbreak of ovine footrot discovered in 2008, was caused by spread of one virulent strain of *D. nodosus* in the county of Rogaland.

All the Norwegian virulent isolates included in this study clustered together in a clonal manner in the dendrogram. Fifty-three of the 54 virulent, serogroup A isolates are likely to represent the outbreak strain or subtypes of this. Thirty-seven of these 53 isolates belonged to one pulsotype (PT 67), while the remaining 16 isolates belonged to 3 PTs that differed from PT 67 by 3 bands or less. The degree of similarity between these 4 PTs indicates that the isolates are closely related, genetically [19]. Although there is no exact documentation of an epidemiological link between all the isolates, they were all from one county with a high density of sheep farms. The combination of the epidemiological data and the phenotypic and genotypic similarites between these isolates in terms of virulence, serogroup, PT and *pgr* variant, strengthens the assumption that they are, indeed, genetically related. Isolates belonging to PT 67 were collected over all 4 years. The remaining 3 PTs were observed in isolates collected in 2010 or 2011. They may represent variants of PT 67 that have undergone a genetic event, which can occur over time. One of the virulent, serogroup A isolates (PT 69) differed only by 2 bands from PT 67, but had the *pgrB* variant. This could be a case of a genetic event involving the *pgr* region, or it may represent a strain not related to the outbreak strain.

Four of the virulent, non-serogroup A isolates differed from PT 67 by 3–4 bands and 2 isolates contained a different *pgr* variant. We believe that these isolates are not genetically related to the outbreak strain. Rather, they may represent virulent strains that were also imported, but did not spread to the same degree. This may be the result of epidemiological circumstances or strain differences in their ability to survive, spread and cause disease under Norwegian conditions.

Three possible cases of serogroup conversion were identified. Virulent, non-serogroup A isolates that were indistinguishable from PT 67 were found in 3 farms where virulent, serogroup A isolates were also found. From one of the farms a benign, serogroup C isolate, a virulent, serogroup C isolate and a virulent, serogroup A isolate were analysed. The 2 virulent isolates were indistinguishable by PFGE. We hypothesise that the benign, serogroup C isolate was the source of a new *fim*A gene to a virulent isolate, but this requires further investigations. Kennan *et al*. [20] demonstrated *in vitro* that serogroup conversion can occur in *D. nodosus* by natural transformation. It is likely that this can also happen under natural conditions. Serogroup conversion may also have occurred on the farms with benign isolates of different serogroups belonging to indistinguishable PTs.

The limited geographical spread of the virulent *D. nodosus* isolates is probably due to the national elimination programme and control measures initiated immediately after the outbreak in 2008 [8]. A general ban on movement of small ruminants between counties, which has been enforced since the 1970s to control maedi-visna and scrapie infection in Norway, is also likely to have contributed.

Results indicate that benign *D. nodosus* are endemic in Norway, and are likely to have been so for a long period of time. These isolates showed considerable genetic diversity and were from sheep, cattle and goats with generally mild disease [6,7]. Although the benign isolates were from 12 of the 19 Norwegian counties, most of them were from Rogaland County. This reflects the relatively few samples from outside Rogaland submitted to our laboratory for culturing of *D. nodosus*. The limited number of isolates from some counties may also be the reason why certain PTs were only found in one county.

Before the outbreak in 2008, the last registered case of ovine footrot in Norway was in 1948 [21]. The restricted movement of animals across counties for the last 40 years means that it is unlikely that the benign strains have spread through trade of live sheep during this period. They must have spread within the sheep population a long time ago, or other routes of transmission exist. Cattle are possible carriers as they are not subject to movement restrictions similar to sheep. In Norway co-grazing of cattle and sheep is practiced, and studies have shown that cross-infection between sheep and cattle may occur [16,22,23].

The correlation between *pgr* variant and the virulence of the *D. nodosus* isolates, as defined by the GG-test, is in agreement with previous findings [12,14]. Our results also strengthen previous suggestions that the *pgrA* gene may be associated with virulence in *D. nodosus* by coding for a protein possibly involved in adhesion to extracelluar matrix [14,24]. Nine isolates were negative for both *pgrA* and *pgrB*, which could indicate that other variants of the *pgr* gene exist.

Two of the Danish *D. nodosus* isolates differed by only 2 bands from the main Norwegian outbreak strain (PT 67). A few sheep imports from Denmark were registered in the years prior to 2008, and included imports to Rogaland County (not published). Sheep imported from Denmark could be the source of the new virulent *D. nodosus* strain, and hence the Norwegian outbreak. However, none of the isolates included in the study were from farms with known export to Norway, and without an epidemiological link it cannot be concluded.

## Conclusions

Genotyping by PFGE shows that the virulent, serogroup A isolates from Rogaland County are genetically similar. This confirms that introduction and local spread of one *D. nodosus* strain caused the outbreak of ovine footrot in Norway in 2008. The fact that this virulent strain has had limited geographical spread increases the likelihood that the outbreak can be controlled and that this strain can be eradicated from Norway.

## Competing interests

The authors declare that they have no competing interests.

## Authors’ contributions

MG contributed to the design of the study, laboratory work (PFGE and *pgr* PCR), analysed the data, and drafted the manuscript. BK performed laboratory work (PFGE) and contributed to the writing of the manuscript. TLL contributed to the design of the study, data interpretation and to writing the manuscript. SV provided epidemiological information and contributed to the writing of the manuscript. HJJ contributed to the design of the study, analysing data and to writing of the manuscript. All authors read and approved the final manuscript.

## References

[B1] Meling S Ulvund MJ Stuen S, Ulvund MJ Flock health visits in 17 sheep flocks in Rogaland Proceedings of the 7th International Sheep Veterinary Congress: 2009; Stavanger, Norway 148 149

[B2] Beveridge WIB Foot-rot in sheep: A transmissible disease due to infection with Fusiformis nodosus (n.sp.). Studies on its cause, epidemiology, and control 1941 Melbourne: Council for Scientific and Industrial Research [Bulletin No. 140]

[B3] Stewart DJ Egerton JR, Yong WK, Riffkin GG Footrot in sheep Footrot and foot abscess of ruminants 1989 Boca Raton, FL: CRC Press 5 45

[B4] Gilhuus M Vatn S Dhungyel OP Tesfamichael B L'Abée-Lund TM Jørgensen HJ Characterisation of *Dichelobacter nodosus* isolates from Norway Vet Microbiol 2013 56 142 148 10.1016/j.vetmic.2012.12.020 23332560

[B5] Palmer MA A gelatin test to detect activity and stability of proteases produced by *Dichelobacter (Bacteroides) nodosus* Vet Microbiol 1993 56 113 122 10.1016/0378-1135(93)90133-R 8236773

[B6] Vatn S Hektoen L Høyland B Reiersen A Kampen AH Jørgensen HJ Elimination of severe footrot from the Norwegian sheep population - a progress report Small Ruminant Res 2012 56 11 13 10.1016/j.smallrumres.2012.04.012

[B7] Vatn S Hektoen L Fredriksen B Høyland B Reiersen A Jørgensen HJ Elimination of footrot in Norway: observed clinical signs in sheep flocks infected with *Dichelobacter nodosus* The 8th International Sheep Veterinary Congress: 2013; Rotorua, NZ: New Zealand Veterinary Association 149

[B8] Vatn S Hektoen L Høyland B Kampen AH Skarra TK Stuen S, Ulvund MJ Surveillance, control and eradication of footrot in Norway Proceedings of the 7th International Sheep Veterinary Congress: 2009; Stavanger, Norway 120 121

[B9] Statistics Norway [https://www.ssb.no/statistikkbanken/SelectVarVal/Define.asp?MainTable=UhArVareLand&KortNavnWeb=muh&PLanguage=0&checked=true]

[B10] Buller NB Ashley P Palmer M Pitman D Richards RB Hampson DJ Understanding the molecular epidemiology of the footrot pathogen, *Dichelobacter nodosus*, to support control and eradication programs J Clin Microbiol 2010 56 877 882 10.1128/JCM.01355-09 20071558 PMC2832437

[B11] Ghimire SC Egerton JR PCR-RFLP of outer membrane proteins gene of *Dichelobacter nodosus*: a new tool in the epidemiology of footrot Epidemiol Infect 1999 56 521 528 10.1017/S0950268899002290 10459657 PMC2809648

[B12] Russell CL Smith EM Calvo-Bado LA Green LE Wellington EMH Medley GF Moore LJ Grogono-Thomas R Multiple locus VNTR analysis highlights that geographical clustering and distribution of *Dichelobacter nodosus*, the causal agent of footrot in sheep, correlates with inter-country movements Infect Genet Evol 2014 56 273 279 23748018 10.1016/j.meegid.2013.05.026 PMC3969714

[B13] Zakaria Z Radu S Sheikh-Omar AR Mutalib AR Joseph PG Rusul G Molecular analysis of *Dichelobacter nodosus* isolated from footrot in sheep in Malaysia Vet Microbiol 1998 56 243 250 10.1016/S0378-1135(98)00219-3 9791871

[B14] Calvo-Bado LA Green LE Medley GF Ul-Hassan A Grogono-Thomas R Buller N Kaler J Russell CL Kennan RM Rood JI Wellington EMH Detection and diversity of a putative novel heterogeneous polymorphic proline-glycine repeat (Pgr) protein in the footrot pathogen *Dichelobacter nodosus* Vet Microbiol 2010 56 358 366 20655152 10.1016/j.vetmic.2010.06.024

[B15] Knappe-Poindecker M Gilhuus M Jensen TK Klitgaard K Larssen RB Fjeldaas T Interdigital dermatitis, heel horn erosion, and digital dermatitis in 14 Norwegian dairy herds J Dairy Sci 2013 56 7617 7629 10.3168/jds.2013-6717 24140335

[B16] Knappe-Poindecker M Gilhuus M Jensen TK Vatn S Jørgensen HJ Fjeldaas T Cross-infection of virulent *Dichelobacter nodosus* between sheep and co-grazing cattle Vet Microbiol 2014 56 375 382 10.1016/j.vetmic.2014.02.044 24698131

[B17] Angen Ø Akkad NB Worm R Nymand A Frosth S Aspán A Slettemeås JS Jørgensen H Klaas IC Detektion af *Dichelobacter nodosus* og sanering for ondartet klovsyge i tre danske fårebesætninger Dansk Vet Tidsskr 2011 56 28 34

[B18] Altman DG Practical statistics for medical research 1991 London: Chapman and Hall

[B19] Tenover FC Arbeit RD Goering RV Mickelsen PA Murray BE Persing DH Swaminathan B Interpreting chromosomal DNA restriction patterns produced by pulsed-field gel electrophoresis: criteria for bacterial strain typing J Clin Microbiol 1995 56 2233 2239 7494007 10.1128/jcm.33.9.2233-2239.1995 PMC228385

[B20] Kennan RM Dhungyel OP Whittington RJ Egerton JR Rood JI Transformation-mediated serogroup conversion of *Dichelobacter nodosus* Vet Microbiol 2003 56 169 178 10.1016/S0378-1135(02)00359-0 12488080

[B21] Øverås J Smittsom klauvsjuke hos sau Et tilbakeblikk på import og utbrudd i Norge. Småfenytt 1994 56 29 33

[B22] Laing EA Egerton JR The occurrence, prevalence and transmission of *Bacteroides nodosus* infection in cattle Res Vet Sci 1978 56 300 304 674842

[B23] Wilkinson FC Egerton JR Dickson J Transmission of *Fusiformis nodosus* infection from cattle to sheep Aust Vet J 1970 56 382 384 10.1111/j.1751-0813.1970.tb15578.x 5471272

[B24] Myers GS Parker D Al-Hasani K Kennan RM Seemann T Ren Q Badger JH Selengut JD Deboy RT Tettelin H Boyce JD McCarl VP Han X Nelson WC Madupu R Mohamoud Y Holley T Fedorova N Khouri H Bottomley SP Whittington RJ Adler B Songer JG Rood JI Paulsen IT Genome sequence and identification of candidate vaccine antigens from the animal pathogen *Dichelobacter nodosus* Nat Biotechnol 2007 56 569 575 10.1038/nbt1302 17468768

